# Supplementary material for: Acute ampakines increase voiding function and coordination in a rat model of SCI
Source: eLife. 2024 Mar 7;12:RP89767. doi: 10.7554/eLife.89767 (PMC10962400; doi:10.7554/eLife.89767)
Supplement: Supplementary file 1. [file elife-89767-supp1.docx]

***Supplemental Table 1.*** Mean data of cystometry measures at 5- days post-injury following HPCD or ampakine CX1739 treatment. Intact (n = 8), SCI (n = 7) groups. Data are presented as Mean ± SD.

| **Group** | **Treatment** | **Threshold**  **(*cmH_2_O*)** | **Intercontraction Interval *(s)*** | **Voided Volume *(μl)*** | **Peak Pressure *(cmH_2_O)*** |
| --- | --- | --- | --- | --- | --- |
| **Intact** | Baseline | 16.1 ± 3.6 | 101.7 ± 37 | 189 ± 56.4 | 29.6 ± 4.5 |
|  | HPCD | 16.2 ± 4.7 | 105.3 ± 27.9 | 206.8 ± 77.6 | 28.3 ± 6.7 |
|  | 5 mg/Kg | 13.7 ± 4.5 | 95.8 ± 25.8 | 178 ± 62.3 | 25.9 ± 5 |
|  | 10 mg/Kg | 12.4 ± 2.7 | 101.6 ± 31.6 | 165.1 ± 62.2 | 23.8 ± 4.3 |
|  | 15 mg/Kg | 12.4 ± 3 | 99.7 ± 24.1 | 173 ± 61.9 | 23.5 ± 3.9 |
| **SCI** | Baseline | 22.2 ± 4.9 | 734.7 ± 322.1 | 1188.3 ± 381.6 | 30.3 ± 8.1 |
|  | HPCD | 21.3 ± 6 | 789 ± 367.3 | 1182.1 ± 467.6 | 27 ± 5 |
|  | 5 mg/Kg | 11.3 ± 3.3 | 504.2 ± 231.6 | 808.9 ± 440.8 | 22.2 ± 5.2 |
|  | 10 mg/Kg | 8.5 ± 1.8 | 303.8 ± 97.4 | 521.3 ± 167.4 | 20.9 ± 7.8 |
|  | 15 mg/Kg | 9.2 ± 4.2 | 340.7 ± 113.1 | 548.6 ± 73.8 | 20.7 ± 8.4 |
