# Supplementary material for: Acute ampakines increase voiding function and coordination in a rat model of SCI
Source: eLife. 2024 Mar 7;12:RP89767. doi: 10.7554/eLife.89767 (PMC10962400; doi:10.7554/eLife.89767)
Supplement: Supplementary file 2. [file elife-89767-supp2.docx]

***Supplemental Table 2.*** Mean data of EUS EMG activity at 5- days post-injury following HPCD or ampakine CX1739 treatment. Intact (n = 8), SCI (n = 7) groups. Data are presented as Mean ± SD.

| **Group** | **Treatment** | **Duration**  **(s)** | **Threshold**  **(*cmH_2_O*)** | **Area under the curve *(a.u.)*** | **RMS_peak_ EMG *(a.u.)*** |
| --- | --- | --- | --- | --- | --- |
| **Intact** | Baseline | 3.0 ± 0.9 | 27.6 ± 3.7 | 0.3 ± 0.2 | 0.1 ± 0.1 |
|  | HPCD | 3.6 ± 1.5 | 24 ± 6.2 | 0.3 ± 0.2 | 0.1 ± 0.1 |
|  | 5 mg/Kg | 3.8 ± 1.6 | 19.7 ± 5.7 | 0.4 ± 0.2 | 0.2 ± 0.2 |
|  | 10 mg/Kg | 3.7 ± 0.7 | 17.1 ± 5 | 0.4 ± 0.2 | 0.1 ± 0.1 |
|  | 15 mg/Kg | 4.2 ± 1.3 | 15.6 ± 2.7 | 0.4 ± 0.3 | 0.1 ± 0.1 |
| **SCI** | Baseline | 10.2 ± 3.9 | 27.4 ± 7.1 | 2.4 ± 1.2 | 0.3 ± 0.3 |
|  | HPCD | 9.4 ± 3.4 | 24.7 ± 6.6 | 2.6 ± 2 | 0.5 ± 0.4 |
|  | 5 mg/Kg | 9.2 ± 3.2 | 17.3 ± 8.8 | 2.2 ± 1.5 | 0.4 ± 0.4 |
|  | 10 mg/Kg | 8.8 ± 2.7 | 11.5 ± 5.2 | 1.6 ± 1.5 | 0.3 ± 0.3 |
|  | 15 mg/Kg | 9.1 ± 1.8 | 9.7 ± 4.4 | 1.5 ± 1.7 | 0.3 ± 0.3 |
